# Predictive Model for Extended-Spectrum β-Lactamase–Producing Bacterial Infections Using Natural Language Processing Technique and Open Data in Intensive Care Unit Environment: Retrospective Observational Study

**DOI:** 10.2196/54044

**Published:** 2024-07-10

**Authors:** Genta Ito, Shuntaro Yada, Shoko Wakamiya, Eiji Aramaki

**Affiliations:** 1 Department of Information Science Nara Institute of Science and Technology Ikoma City Japan

**Keywords:** predictive modeling, MIMIC-3 dataset, natural language processing, NLP, QuickUMLS, named entity recognition, ESBL-producing bacterial infections

## Abstract

**Background:**

Machine learning has advanced medical event prediction, mostly using private data. The public MIMIC-3 (Medical Information Mart for Intensive Care III) data set, which contains detailed data on over 40,000 intensive care unit patients, stands out as it can help develop better models including structured and textual data.

**Objective:**

This study aimed to build and test a machine learning model using the MIMIC-3 data set to determine the effectiveness of information extracted from electronic medical record text using a named entity recognition, specifically QuickUMLS, for predicting important medical events. Using the prediction of extended-spectrum β-lactamase (ESBL)–producing bacterial infections as an example, this study shows how open data sources and simple technology can be useful for making clinically meaningful predictions.

**Methods:**

The MIMIC-3 data set, including demographics, vital signs, laboratory results, and textual data, such as discharge summaries, was used. This study specifically targeted patients diagnosed with *Klebsiella pneumoniae* or *Escherichia coli* infection. Predictions were based on ESBL-producing bacterial standards and the minimum inhibitory concentration criteria. Both the structured data and extracted patient histories were used as predictors. In total, 2 models, an L1-regularized logistic regression model and a LightGBM model, were evaluated using the receiver operating characteristic area under the curve (ROC-AUC) and the precision-recall curve area under the curve (PR-AUC).

**Results:**

Of 46,520 MIMIC-3 patients, 4046 were identified with bacterial cultures, indicating the presence of *K pneumoniae* or *E coli*. After excluding patients who lacked discharge summary text, 3614 patients remained. The L1-penalized model, with variables from only the structured data, displayed a ROC-AUC of 0.646 and a PR-AUC of 0.307. The LightGBM model, combining structured and textual data, achieved a ROC-AUC of 0.707 and a PR-AUC of 0.369. Key contributors to the LightGBM model included patient age, duration since hospital admission, and specific medical history such as diabetes. The structured data-based model showed improved performance compared to the reference models. Performance was further improved when textual medical history was included. Compared to other models predicting drug-resistant bacteria, the results of this study ranked in the middle. Some misidentifications, potentially due to the limitations of QuickUMLS, may have affected the accuracy of the model.

**Conclusions:**

This study successfully developed a predictive model for ESBL-producing bacterial infections using the MIMIC-3 data set, yielding results consistent with existing literature. This model stands out for its transparency and reliance on open data and open-named entity recognition technology. The performance of the model was enhanced using textual information. With advancements in natural language processing tools such as BERT and GPT, the extraction of medical data from text holds substantial potential for future model optimization.

## Introduction

In recent years, machine learning techniques have been used to build models to predict various medical events such as drug-resistant bacterial infections [1] and unscheduled hospital readmissions [2]. Most of these studies have used private data sets to build their prediction models, limiting the replicability and generalizability of the findings owing to accessibility constraints.

In contrast, MIMIC-3 (Medical Information Mart for Intensive Care III) is a large publicly available electronic medical record (EMR) data set that contains comprehensive clinical data of more than 40,000 patients admitted to intensive care units (ICUs), thereby serving as a valuable resource for the development and evaluation of machine learning models for predicting various medical events [3-5]. While previous studies have mainly used structured data such as patient background (eg, age and sex), laboratory results, and vital signs to predict medical events, MIMIC-3 is unique in that it also includes textual data. In a database consisting of a single medical institution, the records of visits to other medical institutions are not structured and may be difficult to trace. However, the text of an EMR may contain records of visits to other hospitals, and if information from such texts can be extracted, even a database obtained from a single medical facility might facilitate the tracking of past medical history. This may also improve the accuracy of predicting medical events because past medical history is often important in predicting such events.

Information extraction from medical texts can be a complex task because of the specialized terminology and abundant abbreviations used. Further, 1 common method to extract information from medical texts is to use named entity recognition (NER), which is a subtask of information extraction that seeks to locate and classify named entities in text into predefined categories, such as the names of diseases, drugs, and medical conditions. For example, QuickUMLS matches strings of text to Unified Medical Language System (UMLS) concepts and extracts concept unique identifiers (CUI) from the text [6]. The UMLS is a comprehensive resource of biomedical terms and concepts that allows QuickUMLS to extract medical information effectively and quickly. QuickUMLS uses a method called “approximate string matching,” which finds UMLS concepts in texts that are either the same or very close to the string in the text.

Among the various medical events that are meaningful to predict, this study focused on predicting infections caused by a type of antibiotic-resistant bacteria known as extended-spectrum β-lactamase (ESBL)–producing bacteria. They are a significant global health concern because of their resistance to commonly used antibiotics [7]. The incidence of ESBL-producing bacteria has been reported to have increased from 1997 to 2011 in the United States [8]. The timely and accurate prediction of ESBL-producing bacterial infections can help initiate appropriate antimicrobial therapy, improve patient outcomes, and minimize the spread of antibiotic resistance.

In this study, using MIMIC-3 as a data source, we constructed and evaluated a machine learning model to predict whether *Escherichia coli* and *Klebsiella pneumoniae* in specimens collected from a patient were suspected of producing ESBLs, based on structured data and patient history information extracted by applying QuickUMLS to EMR text. This study aimed to build a model that makes clinically meaningful predictions using open data sources and open NER technology.

## Methods

### Ethical Considerations

The establishment of the MIMIC-3 database was approved by the institutional review boards (IRBs) of Beth Israel Deaconess Medical Center and the Massachusetts Institute of Technology [4]. Under the Common Rule (45 CFR 46), records-based research using identifiable private information that is publicly available is exempt from IRB review. Since MIMIC-3 is publicly available and deidentified, the secondary analysis in this study is exempt from IRB review. As a result, this study did not undergo an IRB review.

One of the authors (GI) received the necessary training in the use of the MIMIC-3 data set, obtained permission to use the data, and conducted this study in compliance with the PhysioNet Credentialed Health Data Use Agreement 1.5.0.

### Data Source

In this study, we used the MIMIC-3 data set, a publicly available large-scale EMR data set. MIMIC-3 contains comprehensive clinical data from over 40,000 patients admitted to ICUs at the Beth Israel Deaconess Medical Center in Boston, Massachusetts, between 2001 and 2012. The data set includes various types of clinical information such as demographics, vital signs, laboratory results, medications, diagnoses, and free-text data in the form of nursing notes, radiology reports, and discharge summaries [3-5].

### Study Population

This study included patients with *K pneumoniae* or *E coli* detected in bacterial culture tests conducted during hospitalization and detailed in the MIMIC-3. Patients without a summary text at discharge were excluded from this study.

### Outcome Variable

The outcome variable is a binary variable of whether *K pneumoniae* or *E coli* in the specimen showed a minimum inhibitory concentration ≥8 µg/mL for cefpodoxime or minimum inhibitory concentration ≥2 µg/mL for ceftazidime, or ceftriaxone as a result of the bacterial culture test (liquid microdilution method). This criterion corresponds to the screening criteria for ESBL-producing bacteria according to the Clinical and Laboratory Standards Institute M100-S25 [9].

### Predictor Variables

The predictor variables were broadly classified into variables extracted from the structured tables and those related to the patient’s history extracted from the text data.

The variables extracted from the structured table were patient age at the time of specimen collection, sex, number of days between admission and specimen collection, admission type, previous location of the patient before arrival at the hospital, and specimen type collected for the bacterial culture test.

The medical history variables extracted from the discharge summary text were preprocessed using the procedure: (1) from the discharge summary, we extracted paragraphs beginning with the string “Past medical history,” “Past Medical History,” or “PAST MEDICAL HISTORY”; (2) QuickUMLS was applied to the extracted paragraphs to extract the CUI with a Jaccard similarity coefficient of 0.7 or higher; and (3) dummy variables with and without each CUI were used for medical history.

### Model Development and Evaluation

First, we constructed an L1-regularized logistic regression model using only the variables extracted from the structured table. Subsequently, we used the variables derived from the structured table and those related to medical history extracted from the text to build either an L1-regularized logistic regression model or a LightGBM model. We chose L1-regularized logistic regression and LightGBM to build our predictive model primarily for 2 reasons. First, logistic regression is a straightforward linear model used for binary classification, while LightGBM is a more complex model that can handle both linear and nonlinear patterns. This combination allows us to cover a broad range of data behaviors. Second, we used L1 regularization with logistic regression to help manage the model’s complexity by selecting important features. For reference, we constructed a model that judges all positive cases, one that judges all negative cases, and one that judges randomly according to the ratio of positive to negative cases.

The models were constructed and evaluated using stratified group 5-fold cross-validation with patient ID as a group variable. This method ensures an equal class distribution in each fold, while samples from the same patient are not split across different folds. The receiver operating characteristic area under the curve (ROC-AUC) and precision-recall curve area under the curve (PR-AUC) were used to evaluate the performance of each model. The ROC-AUC evaluates the model’s ability to distinguish between classes, whereas the PR-AUC focuses on the model’s performance in terms of precision and recall, which are particularly valuable when dealing with imbalanced data sets [10]. These were calculated for each fold, and the average of the values from each fold was used as the ROC-AUC and PR-AUC of that model.

## Results

### Study Population

The total number of patients registered in the MIMIC-3 database was 46,520, of which 4046 underwent bacterial culture tests, and *K pneumoniae* or *E coli* were detected. After excluding 432 patients without a summary text on discharge, the final study population consisted of 3614 patients. Of the specimens collected from this study’s population, 5272 specimens were positive for *K pneumoniae* or *E coli*, which were the targets of this model.

### Patient Characteristics

Patient characteristics are summarized in Table 1, which shows no notable differences in mean age between negative and positive ESBL screening patients, although there was a slightly higher proportion of older patients (n=1160, 27.3%) aged 80 years or older among negative patients. The sex distribution was slightly higher for females (n=2477, 58.4%) in the negative group and almost the same in the positive group. The average time from admission to specimen collection was 6.1 (SD 11.9) days for negative patients and 12.1 (SD 18.4) days for positive patients. Before arrival at the hospital, the previous location of the patient did not differ markedly between the negative and positive patients. Specimens tested for bacterial growth showed that negative patients had slightly more urine specimens and slightly fewer sputum specimens than positive patients. However, these differences were not statistically significant.

**Table 1:** Patient characteristics.

Characteristics			Patients with ESBL^a^ screening–negative specimen (N=4242)		Patients with ESBL screening–positive specimen (N=1030)
**Age (years)**					
	<20 years, n (%)	10 (0.24)		0 (0)	
	≥20 to <40 years, n (%)	196 (4.62)		64 (6.21)	
	≥40 to <60 years, n (%)	941 (22.18)		275 (26.70)	
	≥60 to <80 years, n (%)	1935 (45.62)		530 (51.46)	
	≥80 years, n (%)	1160 (27.35)		161 (15.63)	
	Mean (SD)	68.8 (15.2)		65.2 (14.5)	
**Sex, n (%)**					
	Male	1765 (41.61)		530 (51.46)	
	Female	2477 (58.39)		500 (48.54)	
Number of days from the admission to specimen collection, mean (SD)			6.1 (11.9)		12.1 (18.4)
**Admission type, n (%)**					
	Emergency	3741 (88.19)		927 (90)	
	Elective	384 (9.05)		76 (7.38)	
	Urgent	114 (2.69)		27 (2.62)	
	Newborn	3 (0.07)		0 (0)	
**Previous location of the patient before arriving at the hospital, n (%)**					
	Emergency department admission	2092 (49.31)		453 (43.98)	
	Clinic referral or premature	920 (21.69)		213 (20.68)	
	Transfer from hospital or extramural	648 (15.28)		208 (20.19)	
	Physician referral or normal delivery	526 (12.40)		116 (11.26)	
	Transfer from a skilled nursing facility	40 (0.94)		29 (2.82)	
	Transfer from other health	15 (0.35)		11 (1.07)	
	Information not available	1 (0.02)		0 (0)	
**Specimen tested for bacterial growth, n (%)**					
	Urine	2395 (56.46)		431 (41.84)	
	Sputum	637 (15.02)		245 (23.79)	
	Blood culture	568 (13.39)		134 (13.01)	
	Swab	197 (4.64)		72 (6.99)	
	Bronchoalveolar lavage	78 (1.84)		18 (1.75)	
	Other	367 (8.65)		130 (12.62)	

^a^ESBL: extended-spectrum β-lactamase.

### Model Performance

**Figure 1:**
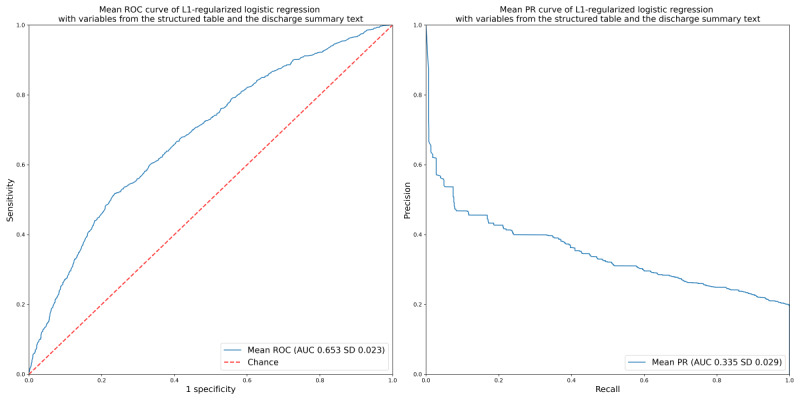
Mean ROC curve and mean PR curve of L1-regularized logistic regression with variables from the structured table and the discharge summary text. The AUC value is presented as the mean (SD) across 5 folds. AUC: area under the curve; PR: precision-recall curve; ROC: receiver operating characteristic.

**Figure 2:**
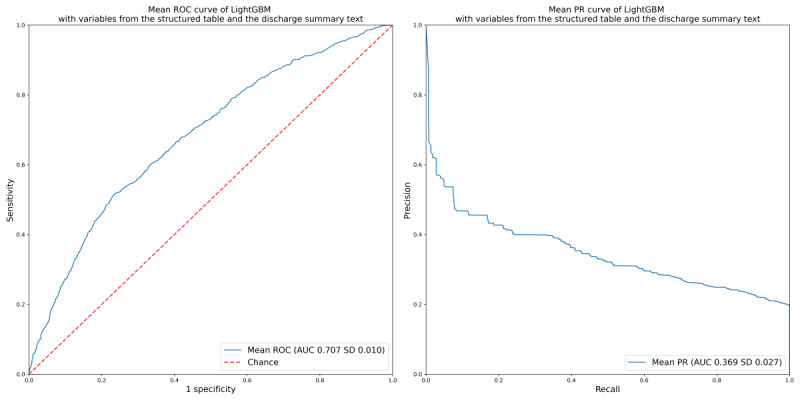
Mean ROC curve and mean PR curve of LightGBM with variables from the structured table and the discharge summary text. The AUC value is presented as the mean (SD) across 5 folds. AUC: area under the curve; PR: precision-recall curve; ROC: receiver operating characteristic.

The ROC-AUC of the L1-penalized model constructed using variables extracted from a structured table was 0.646 and the PR-AUC was 0.307 (Table 2). When variables extracted from the structured table and past medical history variables extracted from the discharge summary were used to construct the L1-penalized model, the ROC-AUC and PR-AUC were 0.653 and 0.335, respectively (Table 2 and Figure 1). The ROC-AUC of the LightGBM model was 0.707 and the PR-AUC was 0.369 (Table 2 and Figure 2). In the reference random classification model, the ROC-AUC and PR-AUC were 0.501 and 0.275, respectively (Table 2). The model that predicted all cases as positive or negative had a ROC-AUC of 0.500 and an undefined PR-AUC (Table 2).

**Table 2:** Model performance.

Predictor variables and model		ROC^a^-AUC^b^	PR^c^-AUC
**Only from the structured table**			
	L1-regularized logistic regression	0.646	0.307
**From the structured table and the discharge summary text**			
	L1-regularized logistic regression	0.653	0.335
	LightGBM	0.707	0.369
**None**			
	Random^d^	0.501	0.275
	All positive	0.500	—^e^
	All negative	0.500	—

^a^ROC: receiver operating characteristic.

^b^AUC: area under the curve.

^c^PR: precision-recall curve.

^d^Random model judges randomly according to the ratio of positive to negative cases.

^e^Not applicable.

### Model Specification

Several key variables related to patient attributes and medical history are highlighted in the feature importance data for the LightGBM model. Specifically, “age at admission” has an importance score of 262, emphasizing its significant predictive value in the model. The “days from hospitalization to specimen collection” feature also plays a critical role with a score of 245.2. Medical conditions such as end-stage renal disease (ESRD) and Pseudomonas infection are also pertinent, with ESRD assigned an importance of 64.4, although the specific score for Pseudomonas infection is not listed in the top features shown. The data further include morbid obesity and diabetes mellitus (DM), which are integral for understanding patient outcomes, though their importance scores need to be specified from the full list. Additional features such as “fright,” “secondary,” “trachy,” and “severed” are less prominent, with “fright” having an importance of 28.7, indicating its relatively lower but still notable influence on the model’s predictions.

## Discussion

### Principal Findings

Compared with the reference random classification model, we observed an improvement in both the ROC-AUC and PR-AUC of our model, built solely on predictor variables from the structured table data. The performance of the model was further enhanced by adding features extracted from text-based medical histories.

A systematic review reported that the ROC-AUCs of existing predictive models without text for drug-resistant bacteria ranged from 0.48 to 0.93 [1]. The performance of our model was neither outstanding nor disappointing; it was somewhere in between. Key features contributing to the model’s performance included age; number of days since hospital admission; and medical history of end-stage renal disease, *Pseudomonas* infection, obesity, and DM. These have been noted in previous studies as risk factors for ESBL-producing bacteria and other drug-resistant bacteria, and it seems reasonable to assume that they are important factors for predicting ESBL-producing bacterial infections [11-15].

While our study’s predictors are consistent with known risk factors for drug-resistant infections, the retrospective nature of our analysis means we might not have fully accounted for all biases inherent in such data. Therefore, our findings do not imply causal relationships and should be interpreted with caution, particularly in clinical applications.

Features from the discharge summary text improved prediction accuracy; however, the feature “fright,” which appears to be unimportant for predicting drug-resistant bacteria, also appeared among the important features. This may be because of the limitations of NER using QuickUMLS. That is, “fright” may have been incorrectly identified because it contains the string “right.” Patients with a long medical history are more likely to include common words such as “right” in their medical history, which may have increased the risk of ESBL-producing bacterial infection due to the long medical history, resulting in a higher feature importance. The accuracy of the history extraction by NER may have led to the extraction of invalid features and prevented the model from reaching a high level of performance.

Another concern is the frequent use of abbreviations in discharge summaries. For example, “DM” is an abbreviation for diabetes mellitus. QuickUMLS performs NER based on string similarity, so the abbreviation “DM” and the full term “diabetes mellitus” are the same, but the strings are not highly similar, making extraction difficult.

Furthermore, the extraction by QuickUMLS of words that might seem less meaningful at first glance, such as “secondary,” “trachy,” and “severed” is another challenge. Specifically, “secondary” is associated with “neoplasm metastasis” (CUI: C0027627), “trachy” with “tracheotomy procedure” (CUI: C0040591), and “severed” with “severing” (CUI: C1306232). However, these words were not always used in the discharge summaries to indicate their corresponding CUIs.

The data used in our study were obtained from MIMIC-3, a single-institution database, and the extraction of information outside the patient’s institution, such as medical history, was possible only through textual information. Medical histories were extracted from the discharge summary texts; however, information such as antibiotic usage history, which is a risk factor for ESBL, may not have been detailed in the discharge summary, leading to potential underextraction.

Additionally, while the MIMIC-III database is open and makes our study results more reproducible, the reliability of its data cannot be fully guaranteed. This means that although our model shows trends similar to previous studies, suggesting it has some validity, the possibility that the model could be invalid cannot be dismissed.

In summary, although feature extraction from the discharge summary texts using QuickUMLS improved the accuracy of predicting ESBL-producing bacterial infections, incomplete data and difficulties in extracting information from the text may have prevented us from extracting all the data required for ESBL prediction. These obstacles may have contributed to the suboptimal performance of the proposed model.

### Conclusions

In conclusion, we constructed a model that predicts ESBL-producing bacterial infections with accuracy comparable to that of previous studies using the publicly available MIMIC-3 data set.

Because our model was constructed using open data and open NER technology, it exhibited a high level of transparency. We believe that this model serves as a valuable reference for future studies in this field.

By extracting information from the text, we enhanced the performance of our model. We posit that if we can extract data from the text with even higher precision, we may be able to further improve the performance of our model.

The advent of transformer-based models, such as BERT and GPT, has led to notable improvements in medical natural language processing tasks [2,16,17]. Given the rise in natural language processing techniques, we believe that further applications for the extraction of information from medical texts, such as those used in our study, are promising.

## Abbreviations

CUI: concept unique identifier
DM: diabetes mellitus
EMR: electronic medical record
ESBL: extended-spectrum β-lactamase
ESRD: end-stage renal disease
ICU: intensive care unit
IRB: institutional review board
MIMIC-3: Medical Information Mart for Intensive Care III
NER: named entity recognition
PR-AUC: precision-recall curve area under the curve
ROC-AUC: receiver operating characteristic area under the curve
UMLS: Unified Medical Language System

## Data Availability

This study used the MIMIC-III database, which is publicly available and contains deidentified data from ICU patients. Access to the data requires the completion of a data use agreement and a training course, available at PhysioNet [19]. The data used in our research adheres to the terms of this agreement, ensuring patient privacy and confidentiality. Researchers can obtain the MIMIC-III data set by following the specified access procedure on PhysioNet.
